# Commissioning neuropsychiatry services: barriers and lessons

**DOI:** 10.1192/pb.bp.114.047290

**Published:** 2015-12

**Authors:** Rahul Bhattacharya, Hugh Rickards, Niruj Agrawal

**Affiliations:** 1 Barts and the London School of Medicine and Dentistry; 2 University of Birmingham; 3 St George's Hospital, London

## Abstract

**Aims and method**

Previous studies have shown variations in commissioning of neuropsychiatry services and this makes access to neuropsychiatric services a post-code lottery. In this survey, we approached all mental health and neuropsychiatric service commissioners within London to map current funding and commissioning arrangements, and explored perceived barriers to neuropsychiatric service commissioning.

**Results**

83% of commissioners within London responded. There was significant variability between neuropsychiatric services commissioned through the mental health stream. Contracting arrangements were variable. Lack of earmarked fund for neuropsychiatry and disjointed funding stream for such services were identified by commissioners as a barrier, as was the critical mass of neuropsychiatric cases.

**Clinical implications**

Neuropsychiatric service development continues to be hindered by lack of clear commissioning process. Strategic drive is needed to promote more equitable neuropsychiatric services. National or regional commissioning covering a large population will provide a better model for neuropsychiatric services to be commissioned.

Neuropsychiatry lies at the interface between neurology and psychiatry.^1^ It is often debated that the narrow focus of neurology and psychiatry services fails to meet the need of patients from a broader neuropsychiatric perspective.^2^ The Cartesian belief of the dualism of mind and body has had repercussions for neuropsychiatric training as well as service provision including commissioning.^3^ Being at the interface of neurosciences, neuropsychiatry services are commissioned through both physical health funding stream and mental health commissioning, though there is lack of clarity around this. This leaves this complex discipline vulnerable to falling between different funding streams.

In the UK, mental health services underwent significant growth under the *National Service Framework* (NSF) initiative for mental health, where the predominant focus was around community services.^4^ Although community models were recommended, neuropsychiatry services remained largely based within academic institutions.^5^ At the height of the NHS expansion, a national survey of neuropsychiatry services revealed them to be ‘patchy’ and ‘grossly inadequate’, and discovered that most of the neuropsychiatry services existed from pre-NSF days.^6^ Historically, neuropsychiatry services developed at certain national or regional centres, such as a national hospital, significantly before NSF or current commissioning arrangements. They continued to serve or were developed at certain regional centres in an *ad hoc* way dependent on local clinical enthusiasm or leadership. At times, such developments were not accompanied by specific commissioning initiatives. There were no systemic drivers or a comprehensive plan to meet the population need. Consequently, patients were often referred ‘out of area’ and services often struggled to meet demand, resulting in long waiting times. The reasons for lack of neuropsychiatry service development in recent years has not been examined, despite increasing recognition of need and demand for neuropsychiatry services and its impact on patients' quality of life. Commissioning arrangements and awareness of neuropsychiatry among commissioners and managers could be one of the reasons behind this.^3^

The aim of this study was to explore commissioning arrangements for neuropsychiatry and perceived barriers for neuropsychiatry commissioning from the perspective of managers responsible for commissioning neuropsychiatry services. We based this study in London as the city is unique in having a significant concentration of neuropsychiatric services along with recognised variability in service provision.^5,7^ At the time of carrying out the study, the Greater London area came under the regional commissioning unit or strategic health authority (SHA), which also made London a regional unit. Therefore, London provided a unit of regional commissioning that could be studied and compared with previous literature. Primary care trusts (PCTs) are local units within the SHA that cover well-defined geographical areas, usually within an administrative unit called a borough. In London there were 31 boroughs and 30 PCTs during our survey. Two boroughs collaborated and acted as one unit.

Apart from mapping current practice, the survey explored perceived barriers for neuropsychiatry service provision from commissioners and providers.

## Method

Two surveys were carried out. One approached all mental health commissioners based at the 30 PCTs in London and the other was a separate survey of neuropsychiatry service providers. See the accompanying paper for details.^10^

All local mental health commissioners (within the PCTs) were contacted electronically with the survey questionnaire and this was followed up by a telephone call. The commissioners were asked whether they were aware of a different route of commissioning neuropsychiatry in their PCT. We were directed to the specialist commissioning unit for neurorehabilitation for the London area and they were contacted for the survey. The specialist neurorehabilitation commissioning forms a non-mental health commissioning route. We were not directed to any other funding or commissioning stream for neuropsychiatry.

The survey mapped commissioning arrangements for neuropsychiatry and provision of services. It was factor-analysed for emerging themes from providers' and commissioner' responses for ‘perception of neuropsychiatry’ and ‘perceived barriers to neuropsychiatry service commissioning’.

## Results

Overall, 30 mental health commissioners from 25 PCTs responded. There was no information from 5 PCTs; response rate for commissioners was therefore 83%. The specialist mental health commissioner for neurorehabilitation for London also responded. There was response from all 9 mental health trusts (specialist mental health providers), although 1 returned an incomplete response; thus, response rate for providers was 100%. Data were also gathered from a tertiary neuropsychiatry centre embedded within an acute hospital trust and one in the voluntary sector catering to patients from London but based outside London. Two independent-sector specialist neurorehabilitation centres were identified and contacted with provider questionnaire, and one of them responded.

The commonest mode of commissioning of neuropsychiatry services was to tertiary services followed by local services. This was followed by ‘national’ services which are essentially tertiary services which are open to referrals from across the country. Funding streams for certain conditions were identified to be other than mental health. Although neuropsychiatry services were tertiary services, they were often also open to direct referral from primary care. Brain injury or neurorehabilitation was commissioned through pan-London specialist commissioning group, i.e. from a ‘specialist’ commissioner across a larger geographical area and services provided by specialist tertiary providers. Commissioning for young-onset dementia and cognitive difficulties was often aligned with other health services for ‘older adults’.

**Fig. 1:**
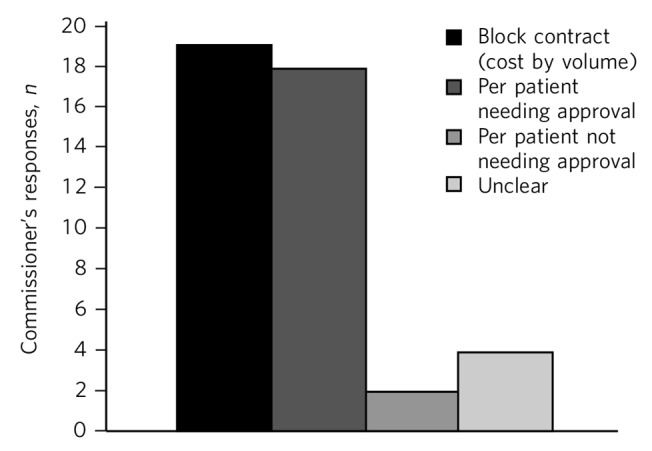
Modes of purchase of neuropsychiatry services.

Figure 1 describes different modes of purchasing neuropsychiatry services. Block contract was the most popular method, closely followed by commissioning per patient but needing approval for all the patients. In only a small minority of cases referrals did not require commissioners' approval. Interestingly, the mode of commissioning was unclear in 4 cases. Some of the commissioners were using more than one method of purchasing neuropsychiatry services.

**Fig. 2:**
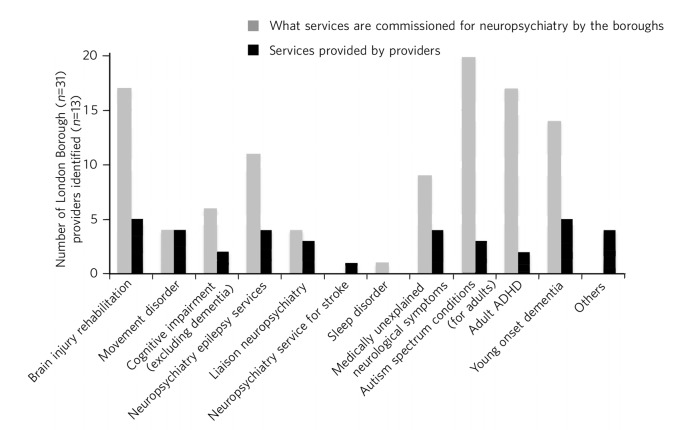
Types of neuropsychiatry services provided and commissioned. ADHD, attention-deficit hyperactivity disorder.

A whole range of neuropsychiatry services were commissioned and provided (Fig. 2). There appears to be a significant disparity in the range of provision and commissioning. This may indicate that some neuropsychiatry services were commissioned as part of a larger service without specific earmarked funding. There was a lack of clarity of commissioning processes in such cases.

Overall, commissioning interest and service provision for specific neuropsychiatric services did not mirror each other. The nine mental health trusts in London were asked about whether they provided neuropsychiatry services. Among them, five identified themselves as providing some form of neuropsychiatry service while four reported not providing any neuropsychiatry service. One of the trusts that no longer provided neuropsychiatry service had a brain injury rehabilitation unit that was closed the year before the survey. There was significant interest in commissioning neurodevelopmental disorders such as autism spectrum and adult attention-deficit hyperactivity disorder (ADHD) (80% and 68% of responding commissioners, respectively), but only 33% of mental health trusts had provision for autism spectrum disorder and 22% provided service for adult ADHD. One of the commissioners reported they commissioned services for sleep disorder but none of the providers had service provision for sleep disorder. Similarly, one of the providers had service provision for stroke-related neuropsychiatry, though none of the commissioners we were able to contact were commissioning such a service.

**Fig. 3:**
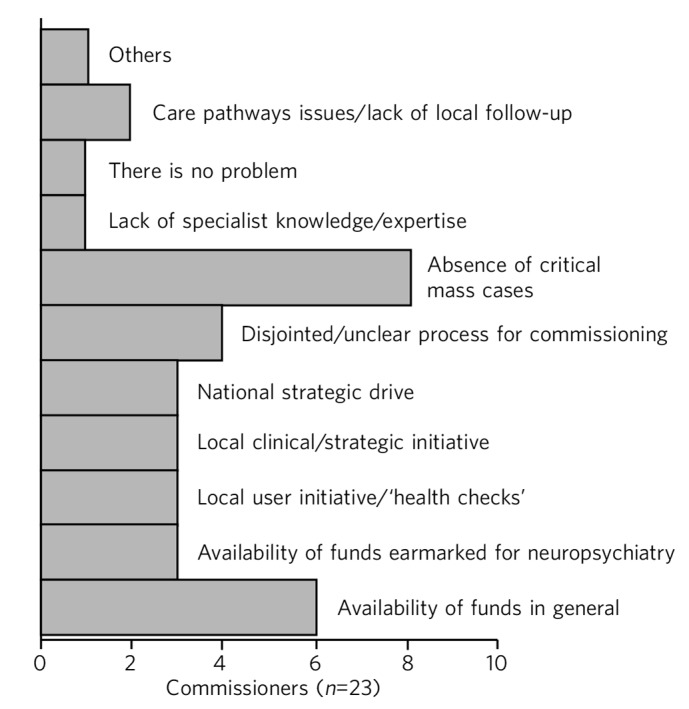
Perceived barriers to neuropsychiatry funding (commissioner perspective).

Both service providers and commissioners identified lack of funds in general and lack of funds specifically earmarked for neuropsychiatry as a barrier to neuropsychiatric service development. Both identified the disjointed nature of commissioning funding streams for neuropsychiatry and lack of national strategic drive for neuropsychiatry as further barriers to commissioning such services. But there were variations in perceptions of commissioners *v.* providers. Of the 30 commissioners who responded, 23 perceived there was a barrier to commissioning neuropsychiatry services (77%). Commissioners were more concerned about lack of critical mass (*n*=8; 35%) of individual neuropsychiatric disorders in their commissioning units or areas (Fig. 3, Box 1).

**Fig. 4:**
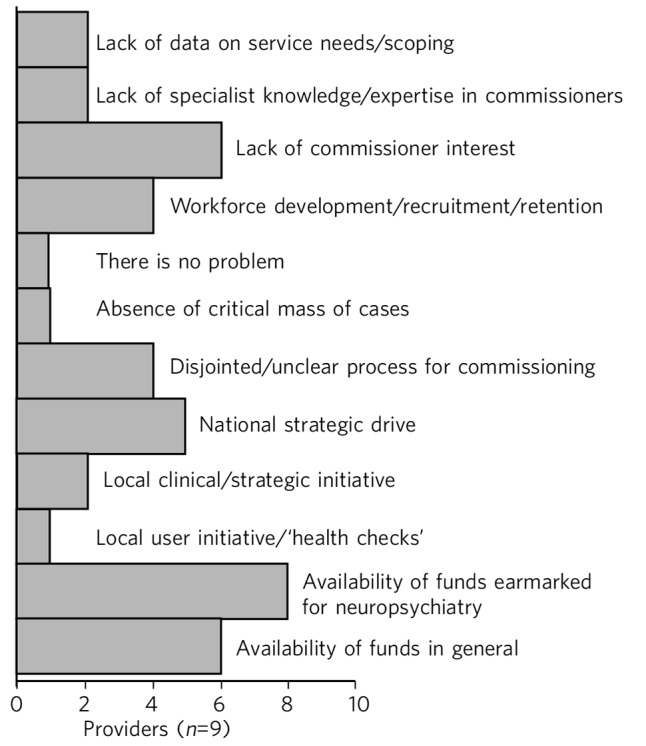
Perceived barriers to neuropsychiatry funding (provider perspective).

Providers often perceived a ‘lack of interest’ in commissioning neuropsychiatric services as a barrier to setting up or providing neuropsychiatry services (Fig. 4).

Commissioners were asked if they were aware of new neuropsychiatry services that were commissioned (expansion) in the past 10 years. There were only 8 responses, suggesting some of the local mental health commissioners were unaware of how services had evolved in the past 10 years locally. We also explored whether there were plans for expansion for neuropsychiatry services locally in the future. From the responses we received, there were services being considered for certain conditions: adult autism spectrum disorder (6; 27%), adult ADHD (4; 18%), young-onset dementia (4; 18%), memory clinic/dementia services (2; 9%), other specific conditions in individual areas (3; 14%); 4 commissioners reported there were no plans to develop new services (18%)

**Box 1** Some comments on neuropsychiatry services from commissioners:

- ‘The numbers of patients requiring these types of services are small in comparison to other psychiatric services and the challenge is therefore being able to provide affordably and locally for this group.’
- ‘Economies of scale for larger areas (e.g. across west London) are needed given that the service may not be sustainable at a borough level’
- ‘The low volume affects local commissioning.’
- ‘Neuropsychiatry is a relatively small field and not high up on the national agenda’.

Two of the nine mental health trusts reported expansion of generic neuropsychiatry services over the past 10 years; two trusts reported no expansion and two other trusts did not know whether there was any expansion. One trust reported there was some patchy development of adult ADHD and young-onset dementia services without commissioning support, largely led by the ‘individual’ interest of clinicians. Another trust also reported patchy (not across all boroughs) development of young-onset dementia, adult autism spectrum disorder and ADHD services. One of the trusts closed down a brain injury rehabilitation service. One of the specialist providers reported developing an adolescent brain injury rehabilitation unit while another reported expansion in the services for medically unexplained neurological symptoms, including in-patient facilities and services for Tourette syndrome.

Commissioners were also asked about future planning in the field of neuropsychiatry services. A third responded that there were no plans for expansion (11/30; 37%). Medically unexplained neurological conditions (along with generic medically unexplained symptoms) were being considered for commissioning in two PCTs while three more PCTs would ‘review’ their current commissioning in this field. Adult ADHD services were being considered in three PCTs while one borough was reviewing their commissioning in the field. Adult autism spectrum disorder service commissioning was being considered by one borough.

Three trusts (one with existing neuropsychiatry services, two without) were unsure whether there would be further expansion of neuropsychiatry services. Two of the mental health trusts had plans of developing generic neuropsychiatry services. One trust reported plans to enhance psychological therapy (cognitive-behavioural therapy) services within the existing neuropsychiatry services. One of the trusts reported plans to develop services for adult ADHD. Two mental health trusts and one of the specialist providers had no further plans to develop neuropsychiatry services in the near future.

## Discussion

This is the first detailed study of commissioners' and providers' views on neuropsychiatry services commissioning. Data were gathered from all the providers and 83% of commissioners, making a robust data-set.

This study shows significant variations and inconsistencies in commissioning of neuropsychiatry services. There is evidence of a disjointed approach towards commissioning neuropsychiatry, despite London being a relatively small geographical area. Certain themes emerged as major barriers which may have contributed to the current state of affairs.

### Critical mass for commissioning neuropsychiatry services

Commissioners identified a lack of ‘critical mass’ as a common barrier to neuropsychiatry service commissioning. Providers also reported this to be a big barrier. In the UK, commissioning is changing radically. As the current structure of purchasing healthcare is reorganised, it can be replaced by a more localised and potentially fragmented system, mirroring the current system. This would be detrimental for neuropsychiatry commissioning as it may mean lower numbers of neuropsychiatry patients per neuropsychiatric condition per commissioning unit and further aggravate the problem of the lack of ‘critical mass’. The barrier of critical mass can be addressed by commissioning neuropsychiatry services for a larger population. We believe a regional or national specialist commissioning panel would best achieve this purpose. This already exists for services such as neurorehabilitation. The specialist commissioning panel mentioned previously reduced variability in neurorehabilitation when compared with borough-based commissioning of neuropsychiatry services.^8^ With the current restructuring of health services in the UK, PCTs and regional commissioning units (SHAs) have been abolished and from 2013 neuropsychiatry is being commissioned by NHS England, although this is going to be reviewed in a few years' time. This might provide commissioners the critical mass to commission neuropsychiatry services more effectively, reduce variability and address unmet needs.

### Knowledge and expertise of commissioners and integrated commissioning

Historically, neuropsychiatry has fallen between neurosciences and mental health commissioning.^9^ The vast majority of providers reported a perceived lack of knowledge and expertise among commissioners, disjointed or unclear commissioning processes, and lack of earmarked funds for neuropsychiatry as challenges to setting up neuropsychiatry services. Commissioners also found a lack of earmarked funds and negotiating multiple funding streams confusing.

Disjointed commissioning and fragmented funding streams without any clear resources earmarked for neuropsychiatry leaves neuropsychiatry at the periphery of multiple streams of funding, for example mental health, older adults' health, neurosciences, specialist neurorehabilitation. It has been hypothesised that there is a lack of adequate understanding of neuropsychiatry among commissioners and service managers, be it of mental health or physical health.^3^ To expect a high level of specialised expertise at every local commissioning unit for a range of neuropsychiatric disorders, each of which have a small local population, is unrealistic. Current restructuring also provides the opportunity for neuropsychiatry services to be commissioned through ‘specialist commissioners’ with earmarked funding. Specialist commissioning covering a substantial geographical area will address concerns of lack of understanding through specialist knowledge as well as ensure there are sufficient patient numbers (critical mass).

From our survey we gathered that very few mental health providers, apart from a few large neuropsychiatry centres, provided care for the vast range of neuropsychiatric conditions. We hypothesise that conditions such as sleep disorder or neuropsychiatric input into neurodegenerative conditions and epilepsy may be closely aligned with acute healthcare, which was possibly not wholly captured in this survey, as they are both far removed from mental health commissioning or mental health trusts and do not have earmarked funding stream that can be reliably traced. There was indirect evidence that the commissioning of these services was possibly linked with generic acute hospitals and funded through physical health funds or a specialist neurosciences funding panel (Fig. 2).

Commissioning in neuropsychiatry needs to be integrated and streamlined. Funding and resources for neuropsychiatry need to be transparent and ring-fenced to allow services to be equitable across the country.

### Need for strategic drive in neuropsychiatry

Commissioners and providers identified a lack of strategic drive as a barrier for neuropsychiatry commissioning. The study shows that where strategic drive exists, even if the condition is rare, it improved standardisation and access to services. The two conditions where this survey found a good degree of shared understanding from provider and commissioning perspectives were brain injury neurorehabilitation and young-onset dementia. Both were supported by the presence of strategic drive, for example the NSF for long-term conditions,^10^ a House of Lords report,^11^ the Department of Health's dementia strategy,^12^ or the National Institute for Health and Care Excellence (NICE) guidelines on dementia.^13^ Services for medically unexplained neurological conditions in London were possibly helped by the recognition of medically unexplained conditions as one of the four streams for which Healthcare for London started working on care pathways in 2008, which later evolved into the Darzi care pathways (the work has been summarised by the report from the Commissioning Support London).^14^ Unlike the NSF for mental health, the NSF for long-term conditions provided an opportunity to foster neuropsychiatric service development.^10^ It was recognised by neuropsychiatrists as a potential strategic driver,^15^ but so far its impact has been arguably limited. Adult ADHD and autism spectrum disorder services were boosted by their respective national clinical guidelines.^16,17^

We believe there is an imminent need for a strategic drive for generic neuropsychiatry, both nationally and internationally. The Royal College of Psychiatrists' working group consensus paper provides an ideal platform to develop strategic drivers to foster neuropsychiatry services' development to meet population needs.^9^

### Developing a shared understanding of what is neuropsychiatry

Different definitions and interpretations of the core neuropsychiatric territory are damaging to the development of neuropsychiatric services globally.^3^ This confusion is not new. In 2005, the International Neuropsychiatric Association identified ‘defining of neuropsychiatry’ as one of the key priorities and ‘first and the most difficult challenge’ to help identifying ‘the legitimate territory of neuropsychiatrist’.^18^ This confusion around the remits of the discipline spills over to neuropsychiatry service provision. There was a significant variation in the familiarity of the different conditions and their commissioning and service provision (Fig. 2).

The perception of what constitutes neuropsychiatry varied significantly among both commissioners and providers. Assessment of local need for commissioning is affected by this uncertainty around prevalence of ‘neuropsychiatric cases’. Through our survey we obtained direct and indirect evidence that neuropsychiatry services were highly non-uniform in what they provided.

It is important to look at neuropsychiatry as a discipline with more clear boundaries and foster development of specific drivers that promote uniform service provision that is both adequate and equitable. Neurodevelopmental disorders (such as autism spectrum disorder and adult ADHD), young-onset dementia and psychiatry of intellectual disability often require skill-sets similar to neuropsychiatry, but traditionally have not been considered its core business. In fact, they do not form part of the core Specialised Services National Definitions Set definition of neuropsychiatry.^19^

We believe the nature of the difficulties seen by neuropsychiatry services is by definition complex and beyond the service provision that could be delivered by either neurology services or mental health services alone. We suggest a basic model with four categories to define the core boundaries of neuropsychiatric disorders (Box 2).

### Limitations

The study was carried out within the Greater London SHA. One can therefore argue that the results might not be generalisable to other areas. However, London was chosen as it had a high concentration of neuropsychiatry centres within a well-circumscribed geographical area located within an SHA where previous service mapping had been carried out.^5,7^ We believe that the problems identified in London can only be an underestimate of commissioning barriers across the country. This can be taken as a pilot study, as information and literature in this field nationally or internationally is very limited.

**Box 2** Core neuropsychiatric disorders

Broadly speaking, neuropsychiatry services provide assessment, investigation and treatment for patients with:

1. a neuropsychiatric disorder (cognitive, behavioural or psychiatric symptoms) associated with a recognised neurological condition or organic brain lesion such as Parkinson's disease, epilepsy, acquired brain injury
2. a neuropsychiatric disorder or mental illness with a yet unrecognised neurological condition or probable organic aetiology (e.g. psychosis related to as yet undiagnosed epilepsy or encephalitis)
3. functional neurological disorders (e.g. dissociative seizures, dissociative memory disorder or conversion disorder) excluding primary presentation with general somatoform disorders without prominent neurological symptoms, chronic fatigue and chronic pain disorders
4. other neuropsychiatric conditions may include specific conditions such as neuropsychiatric sleep disorders, complex neurobehavioural disorders or neuropsychiatric manifestations of extracranial physical conditions.

The study looked into commissioning from the mental health commissioners' perspective and incorporated neurorehabilitation specialist commissioning. However, neuropsychiatry services are located at the interface of neurology and psychiatry and therefore the study may have failed to capture any neuropsychiatry services that are commissioned through and embedded within acute or psychical healthcare setting.

The study surveyed service providers and commissioners and can only comment on the responders' understanding, knowledge and perception of how services were aligned. For the purpose of this study these responses were taken to be proxy measures of the reality of service provision on the ground and the process of their commissioning. The data collected may have been contaminated due to confusion over ‘caseness’ of neuropsychiatry patients.

Neuropsychiatry commissioning remains disjointed and variable. This study identifies barriers for neuropsychiatry commissioning and service development. This makes a case for neuropsychiatry to be commissioned in its entirety through a national specialised commissioning group in the future. This should help to reduce inconsistent provision nationally and help respond to unmet need. There is urgent need for increasing collaborative working between national commissioners and national bodies of neuropsychiatric expertise such as the Royal College of Psychiatrists' Faculty of Neuropsychiatry in the UK. Such a joined-up approach is necessary to develop universally acceptable strategic drives that can foster real improvements in services and benefit patients with neuropsychiatric conditions. We must learn the lessons of the past to break the barriers we continue to encounter.
